# Effects of Mood Inductions by Meal Ambiance and Moderate Alcohol Consumption on Endocannabinoids and *N*-Acylethanolamines in Humans: A Randomized Crossover Trial

**DOI:** 10.1371/journal.pone.0126421

**Published:** 2015-05-11

**Authors:** Ilse C. Schrieks, Dina Ripken, Annette Stafleu, Renger F. Witkamp, Henk F. J. Hendriks

**Affiliations:** 1 The Netherlands Organization for Applied Scientific Research, TNO, Zeist, The Netherlands; 2 Division of Human Nutrition, Wageningen University, Wageningen, The Netherlands; Bielefeld Evangelical Hospital, GERMANY

## Abstract

**Background:**

The endocannabinoid system is suggested to play a regulatory role in mood. However, the response of circulating endocannabinoids (ECs) to mood changes has never been tested in humans. In the present study, we examined the effects of mood changes induced by ambiance and moderate alcohol consumption on plasma ECs 2-arachidonoylglycerol (2-AG), anandamide (AEA), and some *N*-acylethanolamine (NAE) congeners in humans.

**Methods:**

Healthy women (n = 28) participated in a randomized cross-over study. They consumed sparkling white wine (340 mL; 30 g alcohol) or alcohol-free sparkling white wine (340 mL; <2 g alcohol) as part of a standard evening meal in a room with either a pleasant or an unpleasant ambiance.

**Results:**

Plasma concentrations of palmitoylethanolamide (PEA) and stearoylethanolamide (SEA) increased after 30 min in the unpleasant ambiance, while they decreased in the pleasant ambiance. Changes in ECs and their NAE congeners correlated with mood states, such as happiness and fatigue, but in the pleasant ambiance without alcohol only. ECs and their NAE congeners were correlated with serum free fatty acids and cortisol.

**Conclusion:**

This is the first human study to demonstrate that plasma NAEs are responsive to an unpleasant meal ambiance. Furthermore, associations between mood states and ECs and their NAE congeners were observed.

**Trial Registration:**

Clinicaltrials.gov NCT01426022

## Introduction

The endocannabinoid system (ECS) has been suggested to play a role in the regulation of mood [1, 2]. Endocannabinoids (ECs) are endogenous agonists of the cannabinoid receptors CB_1_ and CB_2_. The CB_1_ receptor is located primarily in the brain but has also been identified in peripheral tissues, such as the gastro-intestinal tract and adipose tissue [3]. Anandamide (AEA) and 2-arachidonoylglycerol (2-AG) are the two most well-studied and described ECs. AEA belongs to the *N*-acylethanolamines (NAEs), a group of endogenous compounds that is assumed to share the same biosynthesis and degradation pathways. Unlike AEA, most other NAEs are not able to bind CB_1_ or CB_2_ receptors, but are suggested to be involved in physiological processes via their action on other receptors or via a potentiating (entourage) effect on AEA [4]. NAEs are synthesized from the hydrolysis of their corresponding N- acylphosphatidylethanolamines (NAPEs), whereas 2-AG is produced from diacylglycerol after conversion by diacylglycerol lipase (DAGL). NAEs and 2-AG also have different degradation pathways; NAEs are hydrolysed by fatty acid amide hydrolase (FAAH), while 2-AG is degraded by monoacylglycerol lipase (MAGL) [5].

The effects of AEA and 2-AG on anxiety and depression have been demonstrated in animals. Decreased anxiety and depression responses were demonstrated after inhibition of AEA and 2-AG degradation [6–8] or by microinjection of an AEA analogue into the prefrontal cortex of rats [9]. Human studies also indicate a role of ECs in mood regulation [10–13]. Considerable insight has become available from the use of rimonabant, an inverse CB_1_ agonist. The drug was not approved by the FDA and taken from the market in Europe 1 year after its approval because of side-effects, including anxiety and a depressed mood [10]. Further support for an involvement of AEA and 2-AG in mood comes from a study of Hill et al. (2009) who showed decreased AEA and 2-AG serum levels in individuals with major depression. However, in another study of Hill et al. (2008) individuals with minor depression displayed higher AEA and 2-AG serum levels. These findings suggest that progression of depression might be associated with a reduced ECS activity. Hill and Patel (2013) hypothesized that in healthy individuals the ECS acts as a buffer system that dampens negative emotions to regulate mood, while in individuals with major depression the ECS is hypoactive [11]. This is supported by neuroimaging studies showing a reduced amygdala activity in response to aversive emotional stimuli after cannabinoid administration, or in regular marijuana users [12, 13].

ECs have been demonstrated to have a regulatory role in the stress response as well [14–17]. Mood is negatively influenced by psychological stress, which increases tension and depression. In human interventions, an increase in circulating 2-AG and AEA was shown in response to psychological stress [18, 19]. In the study of Dlugos et al. (2012) also the other NAEs oleoylethanolamide (OEA) and palmitoylethanolamide (PEA) were increased by stress. Therefore, we suggest that NAEs other than AEA may also play a role in mood regulation.

Taken together, these studies suggest that circulating ECs and their NAE congeners are relevant to measure in relation to mood and psychological stress regulation. Circulating concentrations of ECs have been suggested to be a ‘spill-over’ of local production in tissues, as ECs are produced on demand and are thought to have mainly local effects [3, 20]. In a previous study, we showed that plasma AEA and related NAEs strongly correlate with serum free fatty acid (FFA) levels after food intake, suggesting that changes in NAE and FFA concentrations may be induced via common mechanisms [21].

However, to date, no study in humans has been done in which the response of ECs and their NAE congeners on mood changes were evaluated. Mood can be influenced by ambiance both positively and negatively. Ambiance consists of multiple environmental factors, such as lighting and music, which can each affect mood. For example, soft lighting and listening to preferred music makes people feel more comfortable compared to bright lighting and listening to non-preferred music [22]. In addition, mood can be influenced by moderate alcohol consumption. Well-known effects of alcohol on mood are stimulating effects, e.g. feelings of elation and happiness, and increased feelings of relaxation and sleepiness [23–26].

The objective of this study was to examine the effects of mood induction on circulating concentrations of ECs and related NAEs in humans. It was hypothesized that a more negative mood would increase circulating ECs and NAE congeners, whereas a more positive mood would not influence circulating ECs and NAE congeners. Mood was induced by either a positive or negative meal ambiance and by moderate alcohol consumption or no alcohol consumption. Mood was evaluated by a short version of the ‘Profile of Mood States’ (POMS-SF), a validated and widely used mood questionnaire [27]. Plasma concentrations of the ECs 2-AG and AEA and 4 related NAEs were analysed by a validated LC-MS/MS method [28].

## Materials and Methods

### Ethics statement

The study was conducted at TNO (The Netherlands Organization for Applied Scientific Research) in Zeist, The Netherlands, and was performed according to the International Conference on Harmonisation Guidelines for Good Clinical Practice. The study also complied with the Declaration of Helsinki and was approved by an independent ethics committee (METOPP, Medisch-Ethische Toetsing Onderzoek Patiënten en Proefpersonen, Tilburg, The Netherlands). Written informed consent was obtained from all subjects. The study is registered at ClinicalTrials.gov (NCT): NCT01426022. The protocol for this trial and supporting CONSORT checklist are available as supporting information (S1 Protocol and S1 Checklist) [26].

### Subjects

**Fig 1:**
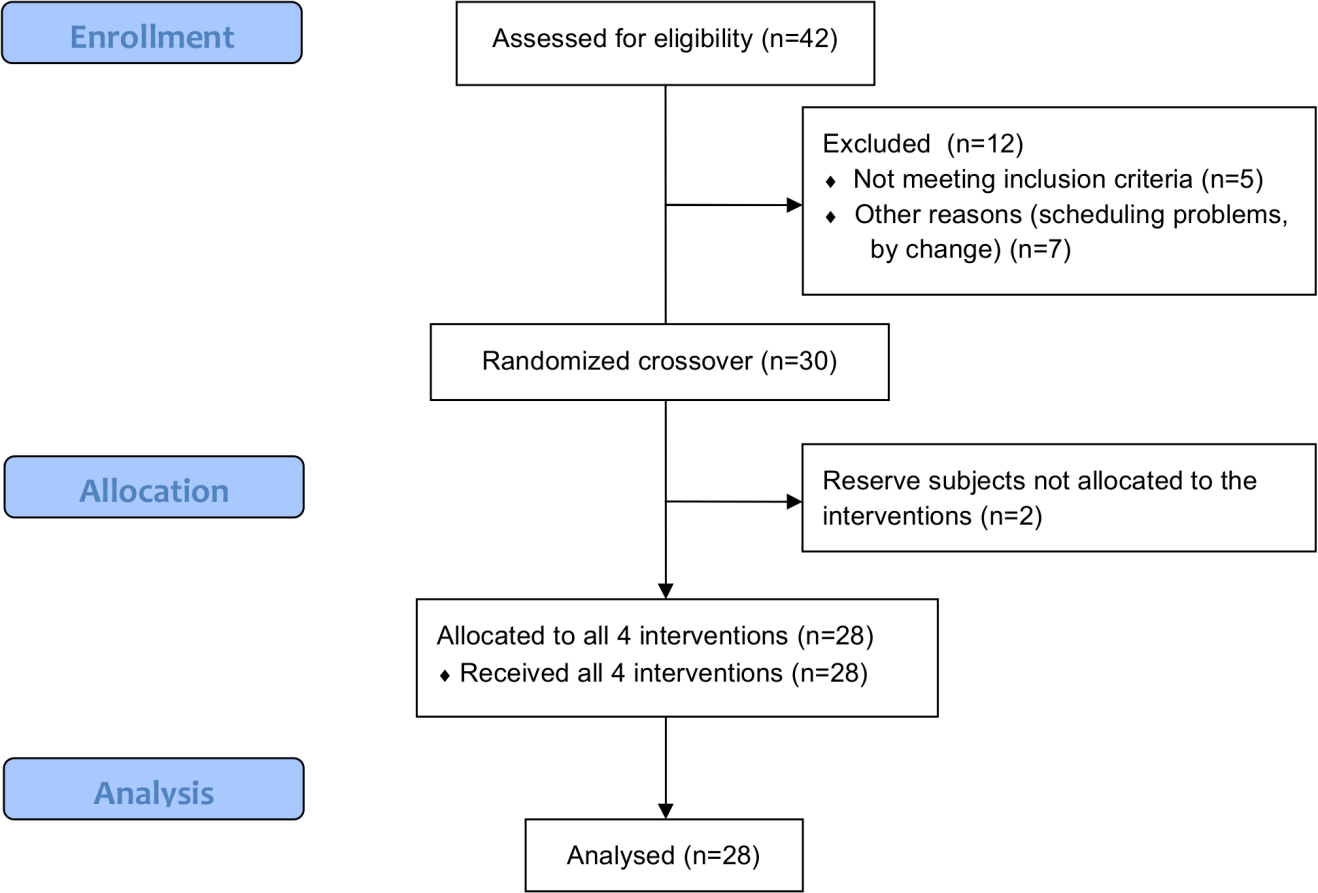
Flow chart (CONSORT). Adapted from Schrieks et al. (2014) [26] under a CC BY license, with permission from PLOS ONE, original copyright 2014.

We recruited 28 non-smoking healthy women within the age of 18–45 years from a pool of volunteers at TNO in Zeist, The Netherlands (Fig 1). Eligible subjects did not use any medication, habitually consumed alcohol (3–20 glasses/week), and had no (family) history of alcoholism. During the screening also the use of THC was excluded. We chose women who were taking oral contraceptives, thus expecting to reduce possible effects of the menstrual cycle on mood. They were not tested in the week they were not taking oral contraceptives. The calculated sample size was 24 subjects, where α was 0.05 (two-sided), β was 0.80, the effect size was 1.2 and the within-person SD was 2.0, based on a previous study with Profile of Mood States (POMS) as outcome measure [29]. This was calculated for a within-subjects analysis of the differences of means in mood (POMS). Twenty-eight subjects were included to guarantee sufficient power, even in case drop out may occur. Furthermore, there were two backup subjects to replace potential early drop out. Subjects were recruited and enrolled in the trial between September and December 2011 (Fig 1).

### Experimental protocol

The study used a randomized, single-blind, crossover design. Subjects consumed three glasses of sparkling white wine (30 g alcohol) or alcohol-free sparkling white wine with a meal in either a pleasant or an unpleasant ambiance. Each subject participated in all four experimental conditions, which occurred at least one week apart. Subjects were equally divided in 4 groups with different intervention orders according to a Latin square design. Allocation to intervention order was randomized according to body fat percentage and age by a computer-generated randomization scheme. Randomization and intervention order allocation were performed by statisticians of TNO. Subjects were kept ignorant to the study aim; they were informed that the study aim was to investigate the effect of different meal settings and alcohol on hormones and satiety. In addition, they were informed the alcohol content of the beverages could vary per intervention day. Subjects were blinded to the alcohol intervention.

**Fig 2:**
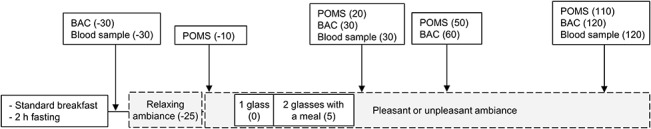
Overview of the experimental procedures during a study day. Time points are indicated within parentheses. BAC, Blood alcohol concentration; POMS, Profile of Mood States questionnaire.

An overview of the procedures during a study day is shown in Fig 2. Subjects were instructed to refrain from drinking alcohol on the preceding evening, to eat their normal breakfast and lunch on standard times, and to refrain from eating and drinking anything except water 2 h before testing. After a baseline blood collection at 16:30 h, subjects relaxed for at least 15 min in a room where soft music was playing. Afterwards, they went to the test rooms with either a pleasant or an unpleasant ambiance. In these rooms, subjects filled out a computer questionnaire on mood before they consumed their first glass of sparkling white wine or alcohol-free white wine at t = 0 within 5 min. Immediately after they consumed their wine, the participants were served a meal consisting of a macaroni dish (2004 kJ, Apetito B.V., Denekamp, The Netherlands) with two more glasses of wine. The meal and 2 glasses of wine had to be finished in 15 min. Blood was drawn by venapuncture before the first drink and 30 min and 120 min afterwards. BAC was measured with a breathalyzer (Alcotest 7410, Dräger Nederland, Zoetermeer, The Netherlands) at regular time points. The mood questionnaire was filled out before the first drink and at 20 min, 50 min and 110 min after.

Minor changes were made in the study procedure after the second study day to reduce potential stress from e.g. number of blood collections. These changes were approved by the Medical Ethics Committee METOPP.

### Mood induction by meal ambiance

Rooms with a pleasant or an unpleasant meal ambiance were created by environmental factors as lighting, music, cleanness, decoration and a film scene. In the pleasant meal ambiance room there was colourful decoration, softened lighting, and music. The unpleasant meal ambiance was created by having very bright lighting, no music, a filled dustbin next to the table, no decoration and plastic cutlery and serving dish. These ambiances were enhanced by showing the participants either a happy or sad film scene from the animation films ‘Bambi’ (Walt Disney, 1942) and ‘The Lion King’ (Walt Disney, 1994). The scenes were approximately 2.5 min long and were shown during the first glass of wine or alcohol-free wine. The happy scenes were either the scene of ‘Bambi on the ice’ or the Lion King scene of ‘hakuna matata’. The sad scenes were the scene of ‘Bambi’s mother dying’ [30] or the ‘Lion Kings father dying’ [31]. Subjects watched each film scene once. Subjects had dinner individually and stayed in the room all the time, except for blood collection.

### Mood induction by alcohol

The mood induction by alcohol consisted of either three glasses of sparkling white wine (30 g alcohol; Prosecco Santa Chiara, Italy) or alcohol-free sparkling white wine (<2 g alcohol; Vita Nova Sparkling Secco, The Netherlands). As alcohol consumption is combined with food consumption, the blood alcohol concentration was expected not to exceed 0.5‰. Therefore, we considered consumption of 30 g alcohol together with a meal as a moderate dose.

### Mood questionnaire

Changes in mood states were measured using the short version of the ‘Profile of Mood States’ (POMS) [27] questionnaire. The questionnaire was computer based and asked participants to answer questions on a five-point interval scale ranging from ‘strongly disagree’ to ‘strongly agree’. The POMS also comprises adjectives for five different subscales for mood. The subscale Anger (range 7–35), Depression (range 8–40), Fatigue (range 6–30) and Tension (range 6–30) refer to a negative mood state, whereas the subscale Vigour (range 5–25) refers to a positive mood state. We added two more positive mood subscales, Happiness (range 4–20) and Calmness (range 4–20) from the Brunel mood scale [32] to make the questionnaire more balanced. Subjects practised the questionnaire once to familiarize.

### Biochemical analyses

Blood samples were collected in tubes containing clot activator for serum or in ice-chilled tubes containing EDTA for plasma. (Vacutainer Systems, Becton Dickinson, Plymouth, UK). For the analysis of ECs and related NAEs, phenylmethanesulfonyl fluoride and URB602 (biphenyl-3-ylcarbamic acid, cyclohexyl ester) were added to the blood samples to inactivate the degradation enzymes fatty acid amide hydrolase and monoglycerol lipase immediately after blood collection. In addition, blood samples for plasma were handled at 4°C and centrifuged within 30 min for 15 min (2000*g*, 4°C) after which they were stored immediately. Serum and plasma samples were stored at -20°C and -80°C respectively, until further analysis. Plasma levels of 2-AG, AEA and the related NAEs, OEA, PEA, stearoylethanolamide (SEA) and docosahexaenoylethanolamide (DHEA) were determined using an LC-MS/MS technique as previously described [28]. Plasma levels of ECs and related NAEs were analysed in a subsample of the subjects (n = 16) that had a complete dataset. Serum concentrations of FFAs and cortisol were determined using Olympus analytical equipment and reagents.

### Data analysis

Statistical analyses were performed using the SAS statistical software package (SAS version 8; SAS Institute, Cary, NC, USA). Data were visually checked on normality and constant variance of residuals with histograms and plots of residuals vs. corresponding predicted values.

Intervention effects were analysed with a mixed analysis of variance model. Because of the crossover design, intervention effects within subjects were compared by including the random factors subject and subject by study day. Alcohol (alcohol vs. alcohol-free), ambiance (pleasant vs. unpleasant ambiance) and time were included as fixed factors. Since the design was a fully crossed design, the two-way interactions between alcohol and ambiance, time and alcohol and time and ambiance, and the three-way interaction between time, alcohol and ambiance were also included as fixed factors in the model. A *post hoc* test with Tukey-Kramer adjustment was used if an intervention effect occurred.

To assess the correlation of mood changes with changes in plasma concentrations of ECs and NAE congeners, Spearman rank correlations were calculated. These correlations were calculated between changes in scores from pre-meal until 110 min after the meal and changes in ECs and NAEs from pre-meal until 120 min after the meal. A Spearman rank correlation was chosen because it is less sensitive to strong outliers than a Pearson correlation.

To assess the correlation of ECs and NAE congeners with FFAs and cortisol, Pearson correlations were calculated for each subject. On these individual correlations a Fisher’s *z* transformation was applied, to correct for deviations from the normal distribution and a 95% confidence interval was calculated. We used this method, because the variation in concentrations was high enough to compute individual correlations, which results in a more powerful calculation of correlations than with Spearman rank correlations.

P values <0.05 were considered statistically significant. The measurements on the first intervention day of the first 11 subjects were considered not valid because of logistic problems that occurred, and were therefore excluded from the analyses. Error bars in figures indicate standard errors of the mean.

## Results

### Subject baseline characteristics

All 28 women completed the study (Fig 1). Subjects’ characteristics are shown in Table 1. During the pre-study screening the Dutch Eating and Behaviour Questionnaire (DEBQ) and State-Trait Anxiety Inventory (STAI) were taken. Subjects had a mean DEBQ restraint score of 2.5 and were therefore interpreted as being non restraint eaters. Their mean score on the STAI trait scale was 33, which is an average score of trait anxiety for women. Highest BAC was observed 30 min after alcohol consumption and was on average (±SD) 0.53 ± 0.09‰.

**Table 1:** Characteristics of 28 women before intervention.

Variable	Mean ± SD	Range
Age (y)	23 ± 5	18–43
Body weight (kg)	66 ± 6	58–79
BMI (kg/m^2^)	22.1 ± 1.7	19.9–26.6
Body fat percentage (%)	24.5 ± 5.5	12.8–33.7
STAI trait score	2.5 ± 0.7	1.1–3.7
DEBQ restrained score	33 ± 6	22–48
Alcohol consumption		
3–6 drinks/week	61%	
7–14 drinks/week	36%	
15–21 drinks/week	4%	

Abbreviations: BMI, body mass index; DEBQ, Dutch Eating Behaviour Questionnaire; STAI, State-Trait Anxiety Inventory.

### Characteristics of the induced mood changes

The mood induction by ambiance influenced tension and depression scores. Tension scores were overall higher in the unpleasant ambiance than in the pleasant ambiance (main effect of ambiance: *P* = 0.018). Depression scores increased in the unpleasant ambiance until 110 min after baseline (*post hoc* tests 110 min vs. -10 min and 20 min: *P* = 0.010 and *P* = 0.008, respectively). The mood induction by alcohol affected calmness and happiness scores. Calmness scores were lower immediately after alcohol consumption as compared to after consumption of alcohol-free drinks (*post hoc* test at 20 min: *P*<0.001). Happiness scores were higher 20 min and 50 min after alcohol consumption than after consumption of alcohol-free drinks (*post hoc* tests: *P*<0.001 and *P* = 0.029, respectively). However, there was also an interaction effect of ambiance and alcohol on happiness scores. The scores were higher in the unpleasant ambiance with alcohol consumption than without alcohol consumption (P<0.001). Happiness scores were not influenced by moderate alcohol consumption in the pleasant ambiance.

### Postprandial responses of ECs and NAE congeners

AEA and related NAEs, with the exception of SEA, changed after a meal (main time effect: all *P*<0.05). *Post hoc* tests showed that OEA and DHEA levels increased 30 min after food intake compared to before the meal (*P* = 0.033 and *P*<0.001, respectively). Additionally, AEA, OEA and PEA concentrations were decreased 120 min after food intake compared to before the meal (*P*<0.001, *P*<0.001, *P* = 0.012, respectively). Furthermore, AEA, OEA and DHEA concentrations were decreased 120 min after food intake compared to 30 min after food intake (all *P*<0.001). Plasma 2-AG concentrations were not influenced by the meal.

### Effects of induced mood changes on ECs and NAE congeners

**Fig 3:**
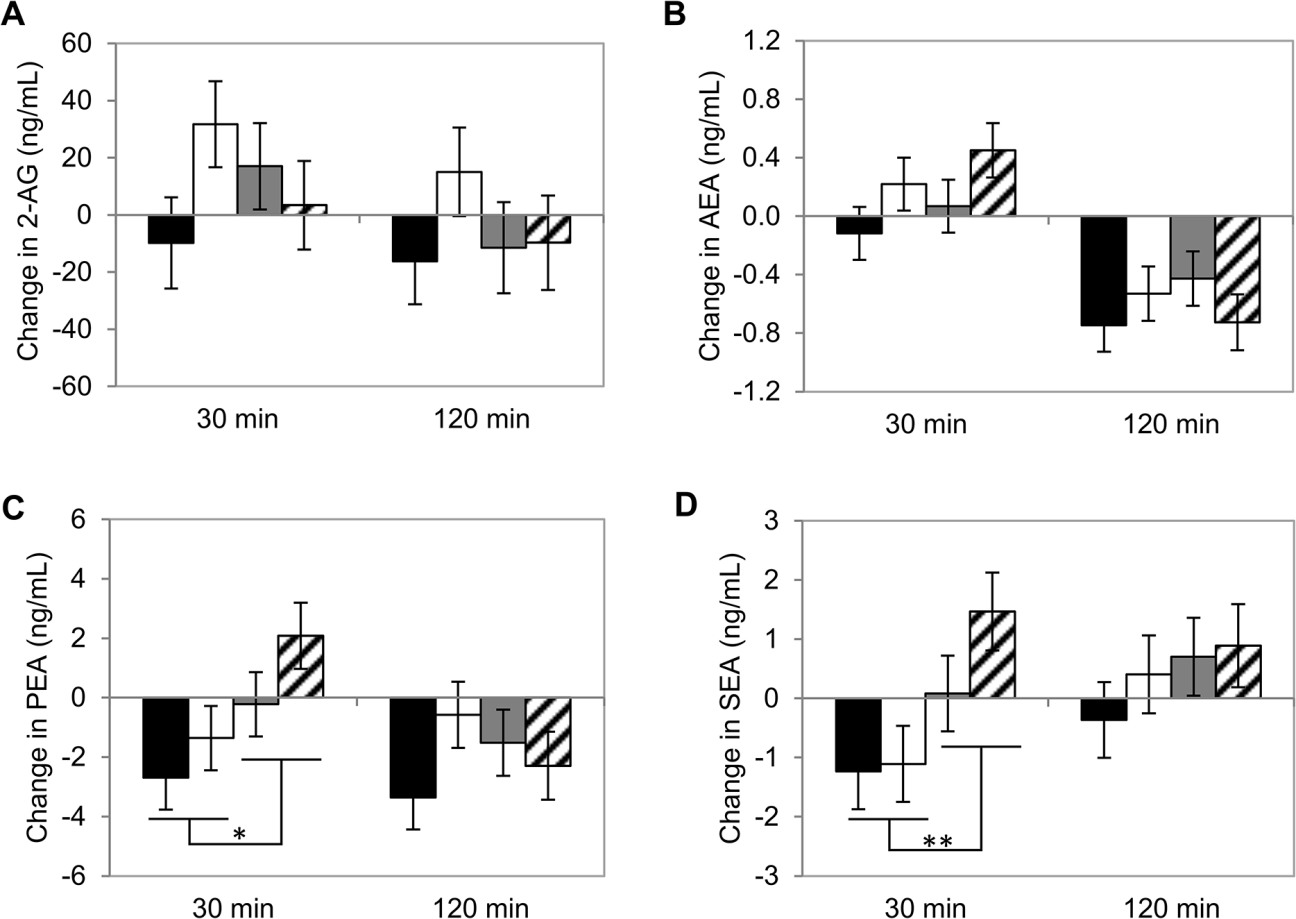
Changes in endocannabinoids and *N*-acylethanolamines after mood inductions by ambiance and moderate alcohol consumption. (A) 2-arachidonoylglycerol (2-AG), (B) anandamide (AEA) and related compounds (C) palmitoylethanolamide (PEA) and (D) stearoylethanolamide (SEA). Black bars represent pleasant ambiance with alcohol; white bars represent pleasant ambiance without alcohol; grey bars represent unpleasant ambiance with alcohol; striped bars represent unpleasant ambiance without alcohol. PEA and SEA concentrations are increased 30 min after a meal in the unpleasant ambiance, but decreased after a meal in the unpleasant ambiance (*P = 0.073; ** *P* = 0.036). n = 16.

The effects of the interventions on the changes in ECs and NAE congeners from baseline were calculated to adjust for baseline differences. In Fig 3 the effect of ambiance on plasma concentrations of ECs and NAE congeners is shown. PEA and SEA concentrations were influenced by ambiance over time (interaction effect of time*ambiance: *P* = 0.041 and *P* = 0.050, respectively). PEA and SEA levels were increased in the unpleasant ambiance but decreased in the pleasant ambiance 30 min after consumption (*post hoc* tests at 30 min: *P* = 0.073 and *P* = 0.036). The ambiance mood induction had no effect on 2-AG, AEA or other NAEs. Furthermore, moderate alcohol consumption did not influence plasma concentrations of 2-AG, AEA or related NAEs.

### Relation with mood states

**Fig 4:**
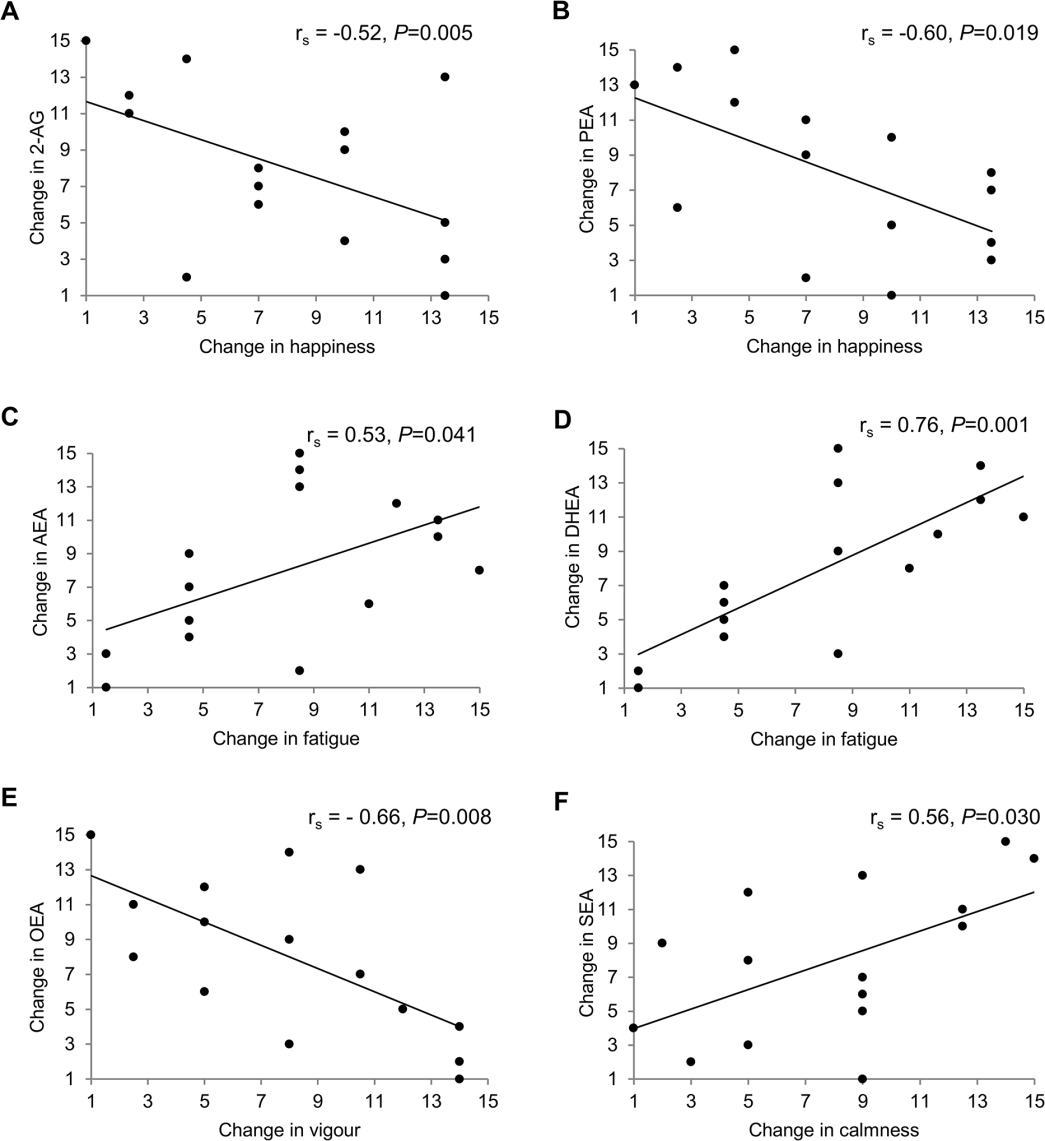
Correlations between changes in mood and endocannabinoids and *N*-acylethanolamines in a pleasant ambiance without alcohol. A) 2-arachidonoylglycerol (2-AG), B) anandamide (AEA), C) palmitoylethanolamide (PEA), D) docosahexaenoylethanolamide (DHEA), E) oleoylethanolamide (OEA), and F) stearoylethanolamide (SEA). The figure shows spearman rank correlations with ranked values on both axes of the panels. n = 16.

Changes in EC and NAE concentrations from baseline until 120 min after the meal correlated with changes in mood states in the pleasant ambiance without alcohol only (Fig 4). Happiness scores were negatively correlated with 2-AG and PEA levels (r_s_ = −0.52 and r_s_ = −0.60, respectively). Furthermore, changes in fatigue scores correlated positively with changes in AEA (r_s_ = 0.53) and DHEA (r_s_ = 0.76), whereas changes in vigour scores correlated negatively with changes in OEA levels (r_s_ = −0.66). Finally, changes in calmness scores correlated positively with SEA levels (r_s_ = 0.56).

### Relation with free fatty acids and cortisol

The postprandial responses of ECs and NAE congeners correlated positively with the postprandial responses of FFAs in all intervention combinations (Table 2). The correlations were not significantly different between the interventions. For SEA, the correlations with FFA change were lower for all interventions as compared with the other NAEs.

**Table 2:** Correlations between endocannabinoid and *N*-acylethanolamines responses and FFA and cortisol responses^1^.

Variable	Intervention	FFA		Cortisol	
		r	(95% CI)	r	95% CI
**2-AG**	Pleasant, no alcohol	0.47	(-0.04 to 0.78)	0.62	(0.19 to 0.86)
	Pleasant, alcohol	0.72	(0.34 to 0.89)	0.54	(0.06 to 0.82)
	Unpleasant, no alcohol	0.82	(0.55 to 0.94)	0.86	(0.64 to 0.95)
	Unpleasant, alcohol	0.49	(-0.01 to 0.79)	0.45	(-0.06 to 0.77)
**AEA**	Pleasant, no alcohol	0.71	(0.34 to 0.89)	0.53	(0.05 to 0.81)
	Pleasant, alcohol	0.73	(0.37 to 0.90)	0.68	(0.27 to 0.88)
	Unpleasant, no alcohol	0.93	(0.82 to 0.98)	0.78	(0.47 to 0.92)
	Unpleasant, alcohol	0.92	(0.77 to 0.97)	0.36	(-0.17 to 0.73)
**OEA**	Pleasant, no alcohol	0.81	(0.53 to 0.93)	0.38	(-0.14 to 0.74)
	Pleasant, alcohol	0.95	(0.87 to 0.98)	0.79	(0.49 to 0.92)
	Unpleasant, no alcohol	0.95	(0.85 to 0.98)	0.82	(0.54 to 0.93)
	Unpleasant, alcohol	0.91	(0.77 to 0.97)	-0.14	(-0.60 to 0.38)
**PEA**	Pleasant, no alcohol	0.74	0.39 to 0.91)	-0.38	(-0.74 to 0.14)
	Pleasant, alcohol	0.72	(0.34 to 0.90)	0.55	(0.07 to 0.82)
	Unpleasant, no alcohol	0.44	(-0.07 to 0.77)	0.31	(-0.22 to 0.70)
	Unpleasant, alcohol	0.82	(0.56 to 0.94)	0.62	(0.18 to 0.85)
**SEA**	Pleasant, no alcohol	0.48	(-0.02 to 0.79)	-0.87	(-0.95 to -0.65)
	Pleasant, alcohol	0.58	(0.12 to 0.84)	0.09	(-0.43 to 0.56)
	Unpleasant, no alcohol	0.47	(-0.03 to 0.79)	0.02	(-0.48 to 0.51)
	Unpleasant, alcohol	-0.23	(-0.65 to 0.30)	-0.51	(-0.80 to -0.01)
**DHEA**	Pleasant, no alcohol	0.84	(0.58 to 0.94)	0.33	(-0.19 to 0.71)
	Pleasant, alcohol	0.76	(0.42 to 0.91)	0.23	(-0.30 to 0.65)
	Unpleasant, no alcohol	0.88	(0.69 to 0.96)	0.60	(0.15 to 0.85)
	Unpleasant, alcohol	0.80	(0.50 to 0.93)	0.18	(-0.35 to 0.62)

^1^ All values are mean [pearson’s correlation coefficient (r)] and 95% confidence interval after Fisher’s *z* transformation (n = 16). Interventions are alcohol (sparkling white wine vs. alcohol-free sparkling white wine) and ambiance (pleasant vs. unpleasant).

Abbreviations: FFA, free fatty acid; NAE, N-acylethanolamine; 2-AG, 2-arachidonoylglycerol; AEA, anandamide; OEA, oleoylethanolamide; PEA, palmitoylethanolamide; SEA, stearoylethanolamide; DHEA, docosahexaenoylethanolamide; DLE, dihomo-γ-linolenoylethanolamide.

The correlations between EC and NAE responses and cortisol responses differed per intervention combination. Plasma concentrations in 2-AG and AEA were positively correlated with serum cortisol concentrations in every intervention combination, except in the unpleasant ambiance with alcohol. OEA and DHEA were also positively correlated with cortisol in the unpleasant ambiance without alcohol (r = 0.82 and r = 0.60, respectively). SEA showed a strong negative correlation with cortisol in the unpleasant ambiance with alcohol (r = -0.87).

**Fig 5:**
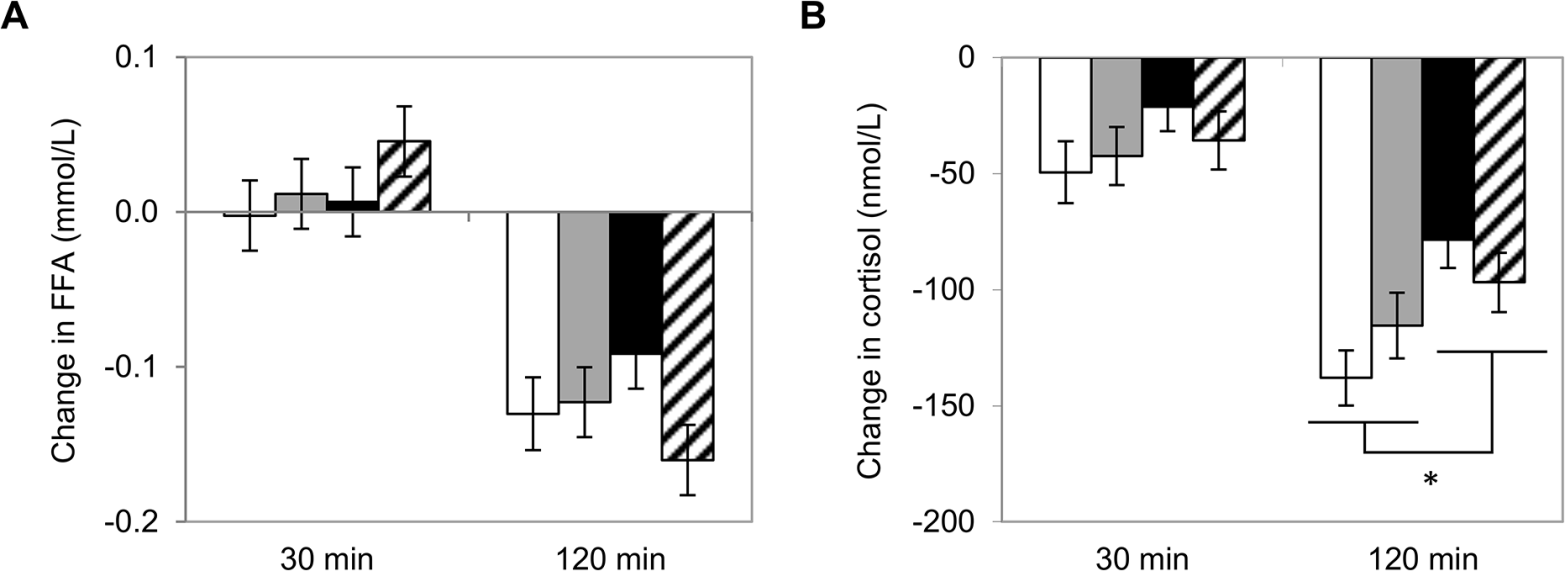
Postprandial changes in (A) serum free fatty acid and (B) cortisol after mood inductions. White bars represent pleasant ambiance with alcohol; grey bars represent unpleasant ambaince with alcohol; black bars represent pleasant ambiance without alcohol; striped bars represent unpleasant ambiance without alcohol. Cortisol concentration is more decreased 120 min after a meal with alcohol than without alcohol (*P = 0.001). n = 28.

The effect of meal ambiance and moderate alcohol consumption on the postprandial responses of FFAs and cortisol are shown in Fig 5. The FFA response was not influenced by the mood inductions. Cortisol was more decreased 120 min after moderate alcohol consumption as compared to after consumption of alcohol-free drinks (*post hoc* test at 120 min: *P* = 0.001).

## Discussion

The primary outcomes of this study are that (1) PEA and SEA were increased in an unpleasant meal ambiance where tension and depression scores were higher; (2) ECs and NAE congeners correlated with mood states in the pleasant ambiance without alcohol only; (3) 2-AG and AEA were not influenced by the mood inductions but were related to FFA and cortisol concentrations.

This study showed that two NAEs, PEA and SEA, were responsive to the mood induction by meal ambiance. In the unpleasant ambiance, PEA and SEA concentrations were elevated after 30 min, while they were decreased in the pleasant ambiance. Possibly, these NAEs can become elevated in a tension and depression increasing ambiance, as scores for tension and depression were higher in the unpleasant ambiance. This concords with animal studies indicating a regulatory role for ECs and related NAEs in emotion and stress [16, 33]. In addition, this would be in line with human studies showing elevated circulating PEA during psychological stress and elevated PEA and SEA in patients with posttraumatic stress disorder [19, 34]. Little is known about the role of PEA and SEA in mood, although PEA has been investigated for its anti-inflammatory, neuroprotective and analgesic properties [35–37].

We investigated whether patterns of correlations existed between postprandial mood responses and circulating ECs and their NAE congeners. In the pleasant ambiance without alcohol we found negative correlations with happiness (2-AG and PEA) and vigour (OEA) and positive correlations with fatigue (AEA and DHEA). These findings might suggest that increasing ECs and NAE congeners are related to decreasing mood states as we hypothesized. However, calmness was positively related to SEA concentrations and no correlations were not found in the other experimental conditions. Therefore, we conclude that we did not observe a consistent pattern of correlations.

The biological relevance of plasma concentrations of ECs and NAE congeners remains speculative because plasma levels are likely to reflect a ‘spill over’ of 2-AG and NAE synthesis in tissues, such as adipose tissue, liver and perhaps brain. Joosten et al. (2010) observed that circulating FFAs and NAEs are highly correlated. Here, correlations were calculated between NAEs and FFA levels similarly as in Joosten et al. (2010) and similar correlations were found between postprandial responses of FFAs and AEA, OEA and PEA. Therefore, this study confirms that plasma NAE patterns may reflect fatty acids patterns and that their short-term release from *N*-acylphosphatidylethanolamine (NAPE) is triggered by similar mechanisms as are involved in the release of FFA. However, in this study, plasma ECs and related NAEs were also positively related to serum cortisol concentrations, especially in the unpleasant ambiance without alcohol. This finding together with the results from previous human studies where ECs and NAE congeners are shown to be responsive to stress [18, 19, 38], supports a role for plasma ECs and NAE congeners as indicators of stress.

Strengths of the study are the randomized crossover design and the controlled study conditions. Furthermore, the mood induction method by meal ambiance was completely standardized and induced a positive and negative mood for a relative long time of three hours. This was shown by higher depression and tension scores in the unpleasant ambiance and higher happiness scores in the pleasant ambiance. In comparison with the mood induction by ambiance, moderate alcohol consumption influenced different mood states and effects were more acute. We also showed an interaction effect of alcohol and ambiance on happiness, which was not shown before. Moderate alcohol consumption in an unpleasant ambiance increased happiness, but drinking alcohol during a pleasant mood results in an equally positive mood state. This indicates that the mood induction methods used were complementary and interacting, thereby providing the ability to study the response of ECs and NAE congeners on a variety of mood changes.

This study also has some important limitations. The experimental design of this study may not have provided the appropriate conditions to examine the relation between changes in mood and changes in ECs and related NAEs. A further limitation of the study is that ECs and their NAE congeners were measured at three time points and the effects of ambiance on NAEs may have been missed. Additionally, mood was induced by rooms with a pleasant or an unpleasant ambiance. Perhaps, these ambiances did not influence mood to such an extent that correlations between mood scores and plasma ECs and NAE congeners could be observed. Therefore, further research may focus on mood induction by ambiance using more extreme conditions. The role of ECs and NAE congeners may also be better detectable when no food is consumed during mood induction, and consequently exclusively focusing on the association of mood with ECs and NAE congeners.

This study was carried out in women, and generalizability of the results to men may therefore not be directly possible, because men and women showed different mood effects of a meal in a previous study [39].

We conclude that this is the first human study to demonstrate that plasma NAEs are responsive to an unpleasant meal ambiance. Furthermore, associations between mood states and ECs and related NAEs were observed, but not in all intervention combinations. This study provides additional insight in the response of plasma ECs and NAE congeners on mood changing conditions in humans.

The findings are in line with animal and human studies suggesting a role of endocannabinoids in mood and stress regulation. However, for a better translation between animal and human studies, animal studies should also measure circulating levels of ECs and NAE congeners and study their response on mood inductions. This would provide more information on the clinical relevance of changes in circulating ECs and NAEs. For clinical implications on the use of plasma ECs and NAE congeners as diagnostic agents for mood disorders, more research is necessary. Future human studies may focus on mood induction by ambiance using more extreme conditions. Associations may also be better detectable when no food is consumed during mood induction, and consequently exclusively focusing on the association of mood with endocannabinoids and NAEs.

## Supporting Information

S1 Checklist CONSORT checklist of information to include when reporting a randomised trial. (DOC)

S1 Protocol Study protocol “Effect of moderate alcohol consumption on postprandial mood.” (PDF)
